# Comparative Analysis of Muscle Hypertrophy Models Reveals Divergent Gene Transcription Profiles and Points to Translational Regulation of Muscle Growth through Increased mTOR Signaling

**DOI:** 10.3389/fphys.2017.00968

**Published:** 2017-12-04

**Authors:** Marcelo G. Pereira, Kenneth A. Dyar, Leonardo Nogara, Francesca Solagna, Manuela Marabita, Martina Baraldo, Francesco Chemello, Elena Germinario, Vanina Romanello, Hendrik Nolte, Bert Blaauw

**Affiliations:** ^1^Venetian Institute of Molecular Medicine, Padova, Italy; ^2^Department of Biomedical Sciences, University of Padova, Padova, Italy; ^3^Molecular Endocrinology, Institute for Diabetes and Obesity, Helmholtz Diabetes Center and German Center for Diabetes Research, Neuherberg, Germany; ^4^Department of Biology, University of Padova, Padova, Italy; ^5^Institute for Genetics, Cologne Excellence Cluster on Cellular Stress Responses in Aging-Associated Diseases, University of Cologne, Cologne, Germany

**Keywords:** mTORC1, skeletal muscle, hypertrophy, ribosome biogenesis, immediate early genes, overload, postnatal growth, Akt

## Abstract

Skeletal muscle mass is a result of the balance between protein breakdown and protein synthesis. It has been shown that multiple conditions of muscle atrophy are characterized by the common regulation of a specific set of genes, termed atrogenes. It is not known whether various models of muscle hypertrophy are similarly regulated by a common transcriptional program. Here, we characterized gene expression changes in three different conditions of muscle growth, examining each condition during acute and chronic phases. Specifically, we compared the transcriptome of Extensor Digitorum Longus (EDL) muscles collected (1) during the rapid phase of postnatal growth at 2 and 4 weeks of age, (2) 24 h or 3 weeks after constitutive activation of AKT, and (3) 24 h or 3 weeks after overload hypertrophy caused by tenotomy of the Tibialis Anterior muscle. We observed an important overlap between significantly regulated genes when comparing each single condition at the two different timepoints. Furthermore, examining the transcriptional changes occurring 24 h after a hypertrophic stimulus, we identify an important role for genes linked to a stress response, despite the absence of muscle damage in the AKT model. However, when we compared all different growth conditions, we did not find a common transcriptional fingerprint. On the other hand, all conditions showed a marked increase in mTORC1 signaling and increased ribosome biogenesis, suggesting that muscle growth is characterized more by translational, than transcriptional regulation.

## Introduction

The regulation of skeletal muscle mass reflects changes in protein synthesis and protein degradation. During various catabolic conditions like aging, denervation, and starvation, increases in protein degradation lead to muscle atrophy. More than a decade ago it was shown that muscle atrophy occurring in these different conditions shows similar transcriptional adaptations (Lecker et al., 2004). They identified genes that increased or decreased in various conditions of muscle atrophy (so-called atrogenes), and further in-depth investigations of their importance have greatly contributed to the discovery of new mechanisms and factors involved in protein degradation.

On the other hand, stretching, high-intensity exercise, or exposure to certain hormones leads to increases in protein synthesis and subsequent muscle hypertrophy. Physiological muscle growth, in which an increase in muscle mass is accompanied by an increase in muscle force, is due to an increase in size of the existing fibers and can be accompanied by addition of new nuclei to the growing fibers. How muscle growth is regulated in different conditions is still a relatively open question. It has been established that one of the major pathways regulating adult muscle mass is the IGF-1-Akt-mTORC1 pathway, which is thought to act mainly through increases in protein synthesis by modulating translation initiation (Manning and Toker, 2017). Despite this important role for protein translation in muscle hypertrophy, there are various examples in which transcription factors regulate adult muscle mass. Overexpression of the transcription factor JunB is sufficient to induce a 40% fiber growth, and this hypertrophy is independent-and additive to Akt-induced fiber growth (Raffaello et al., 2010). Furthermore, the other main pathway regulating adult muscle mass, i.e., the myostatin pathway, also depends on the activity of two transcription factors, smad2 and smad3. Knock down of these two transcription factors leads to muscle hypertrophy which is only partially reduced by the allosteric mTOR-inhibitor rapamycin. Recently, also a role for the transcriptional co-activator PGC1-α4 (Ruas et al., 2012; Mammucari et al., 2015), and the muscle-specific transcription factor MRF4, was suggested to stimulate adult muscle hypertrophy. These results show that muscle growth can be regulated by transcriptional mechanisms, which do not always require full activation of mTORC1 signaling. While some studies have examined changes in the transcriptome during different conditions of muscle hypertrophy (Chaillou et al., 2013, 2015; Barbé et al., 2017), a comparative analysis of the transcriptional changes in various models of muscle growth is still missing.

Here, using microarray analysis, we compared the transcriptional profiles of three different models of functional muscle growth in order to identify the commonly up-or down-regulated genes, i.e., hypertrogenes. We examined the gene-expression profiles of EDL muscles during postnatal (PN) growth (both 2 and 4 week old mice), functional overload (OL) (after 24 h and 3 weeks), and Akt overexpression (24 h and 3 weeks). These experimental models allowed us to identify hypertrogenes during different conditions of muscle growth. Importantly, all models which were chosen show increases in muscle mass which is accompanied by an increase in muscle force.

An important open issue in functional muscle growth is the requirement of satellite cell activation and incorporation. While it has been shown that muscle growth can occur without satellite cell addition (Blaauw et al., 2009; Murach et al., 2017), there is also evidence that deletion of satellite cells significantly reduces or even blunt muscle growth (Egner et al., 2016; Murach et al., 2017). Here, we examined the transcriptional profile of muscle growth models undergoing active proliferation and incorporation of satellite cells (2 week old mice, functional overload) together with those which show a significant reduction, or even absence, of proliferating satellite cells (4 week old mice, Akt overexpression) (Blaauw et al., 2009; White et al., 2010). In addition we evaluated the differences in time courses, examining models of early (24 h) or late (3 weeks) phases of muscle growth.

## Materials and methods

### Models of muscle growth

In this study, we decided to focus on the extensor digitorum longus (EDL) muscle. The choice for this muscle was based on the fact that the functional overload was done by performing a tenotomy of the tibialis anterior tendon, as described by others (Bruusgaard et al., 2010). Briefly, mice were anesthetized and the tendon of the tibialis anterior (TA) was cut and sutured back onto the body of the TA muscle. This procedure exposed only 20–30% of the EDL muscle and is less invasive than the very drastic synergist ablation model, which leads to an overload of the plantaris muscle. The strain of mice used for all groups was determined by the strain of the Akt transgenic mice (Blaauw et al., 2009). The Akt transgenic mouse is generated by crossing a transgenic line which expresses the Cre recombinase under a muscle-specific promoter (Bothe et al., 2000) with a second line which expresses Akt only after the deletion of an upstream DNA sequence by the Cre recombinase (Kroll et al., 2003). The myristolated form of Akt is bound to an estrogen receptor domain, thereby requiring tamoxifen for its stabilization and activation. Muscles were analyzed either 24 h after one single injection of tamoxifen (1mg), or after 3 weeks of tamoxifen treatment once every other day. As the Akt transgene is silent without tamoxifen, we used non treated Akt mice of 2, 4 weeks, and 3 months old as respectively the postnatal 2 weeks, 4 weeks, and control group. All muscles were collected at 9 a.m. to avoid differences due to divergent circadian rhythms and activity patterns (Dyar et al., 2015).

### Gene expression profiling and analyses

Total RNA was isolated using TRIzol (Invitrogen) followed by cleanup with the RNeasy Mini Kit (Qiagen). RNA integrity was evaluated with the Agilent 2100 Bioanalyzer (Agilent Technologies, Palo Alto, CA) and quantified with a NanoVue spectrophotometer (GE Healthcare Life Sciences, Baie d'Urfe, QC). For gene expression profiling, 250ng of RNA from EDL muscles was hybridized to Mouse Gene 1.0 ST Arrays (Affymetrix) using four biological replicates for each growth condition. Expression values were generated from fluorescence signals using the robust multi-array average procedure (RMA) (Irizarry et al., 2003). Specifically, intensity levels have been background adjusted, normalized using quantile normalization, and log2 expression values calculated using median polish summarization and Entrez custom chip definition files for mouse arrays (version 14.1.0) (Dai et al., 2005).

To identify significantly different expressed genes, p-values were calculated by a two-sided *t*-test based on log2 transformed intensities. To correct for multiple testing a permutation-based approach described by Tusher et al. (2001) was used (500 permutations and a fudge factor s0 of 0.1). Gene ontology annotations were added based on the Uniport Database. The 1D and 2D-enrichment tool was utilized to find enriched GO terms along log2 ratios of each condition (Cox and Mann, 2012). For visualization and principal component analysis (PCA) we used an in-house developed tool (InstantClue).

### Western blotting

Western blotting analyses were performed as described previously (Marabita et al., 2016). Antibodies for P-Akt, Akt, eIF4E, P-eIF4E, P-eIF4B, eIF4B, eIF4A, eIF4H, eIF4G, P-eIF4G, 4E-BP1, P-S6, S6 were taken from Cell Signaling. Actin was from Santa Cruz, and puromycin from Millipore. All quantifications of the western blots were done on at least four different blots for each protein, in each condition. Differences between groups were assessed using Student's *t*-test. Significance was defined as a value of *P* < 0.05 (95% confidence).

## Results

### Multiple models of muscle growth are characterized by an increase in puromycin incorporation

**Figure 1:**
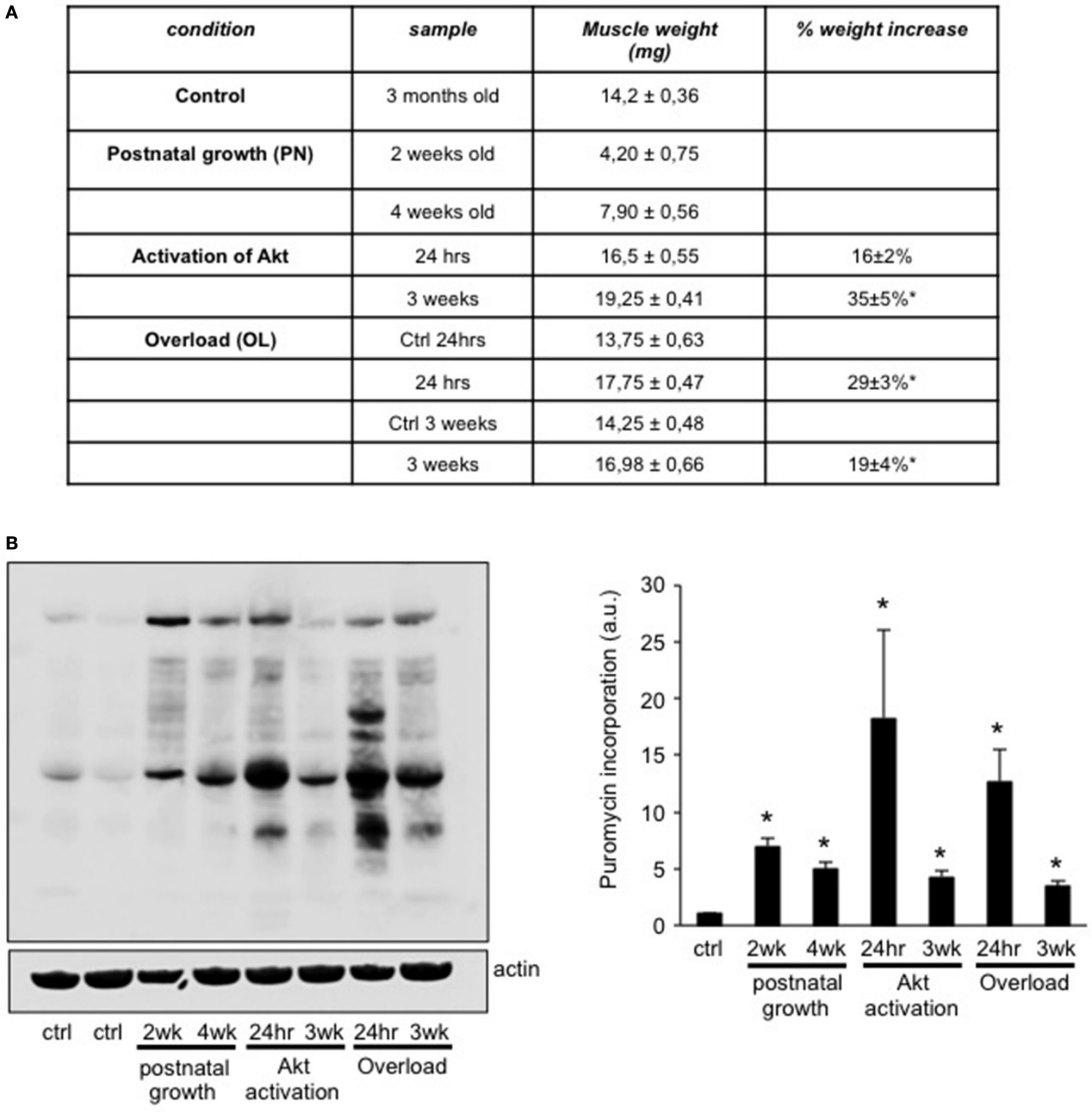
Different models of muscle growth show an increase in protein synthesis rates. **(A)** Table summarizing the weight of the EDL muscles taken out in the six different conditions of muscle growth and control muscles. **(B)** In order to determine the protein synthesis rate in each EDL muscle in the different conditions we injected puromycin 30 min before taking out the muscles. As can be seen in the representative blot and quantification, all conditions show a significant increase in protein synthesis rates compared to control muscles (*n* = 4 for each group, ^*^P < 0.05).

Various factors can increase muscle size, like stimulation with anabolic hormones, or increases of the mechanical load placed on the muscles (Blaauw et al., 2013). In order to understand if muscle hypertrophy during different conditions depends on a common transcriptional profile, we compared the transcriptome of three different conditions of muscle growth; i.e., postnatal growth, mechanical overload by tenotomy, and hyperactivation of Akt only in skeletal muscle. We chose two different time points of postnatal growth, namely EDL muscles taken out at 2 and 4 weeks of age. These time points were chosen as in both cases muscle fibers go through a pronounced increase in fiber size, but the number of myonuclei significantly increases only until 3 weeks of age (White et al., 2010). In the mechanical overload of the EDL muscle we used a tenotomy of the Tibialis Anterior (TA) muscle, which is known to lead to a significant hypertrophy with less damage than the very drastic synergist ablation model (Bruusgaard et al., 2010). As can be seen in Figure 1A, 24 h after tenotomy muscles were already 29 ± 3% bigger than the contralateral EDL. This very rapid increase in muscle mass can largely be attributed to edema, as shown by the space between the fibers found in the H&E staining (Supplementary Figure 1). This drastic initial increase in muscle weight leads to a 19 ± 4% increase in lean muscle mass 3 weeks after the tenotomy, without any signs of edema. The last model of muscle growth we considered is one where we can activate a myrostilated form of Akt only in skeletal muscle. We have previously shown that treatment of this transgenic mouse with tamoxifen leads to a rapid increase in muscle mass, which is accompanied by an increase in force 3 weeks after Akt activation (Blaauw et al., 2009). In order to distinguish between the early induction phase of hypertrophy and the later, more stable phase, we considered also here the effect of Akt activation after 24 h and 3 weeks.

In order to determine the rate of protein synthesis in the various models of muscle growth we injected the antibiotic puromycin in the different conditions. Puromycin incorporation is a reliable index of protein synthesis rate (Goodman et al., 2011) and we have previously found that this it corresponds closely with increased muscle mass (Marabita et al., 2016). As can be seen in Figures 1B, all conditions examined show a significant increase in puromycin incorporation as compared to control mice, with the most pronounced increases observed 24 h after Akt activation or tenotomy.

### Major differences in transcriptional regulation during muscle growth in various models

**Figure 2:**
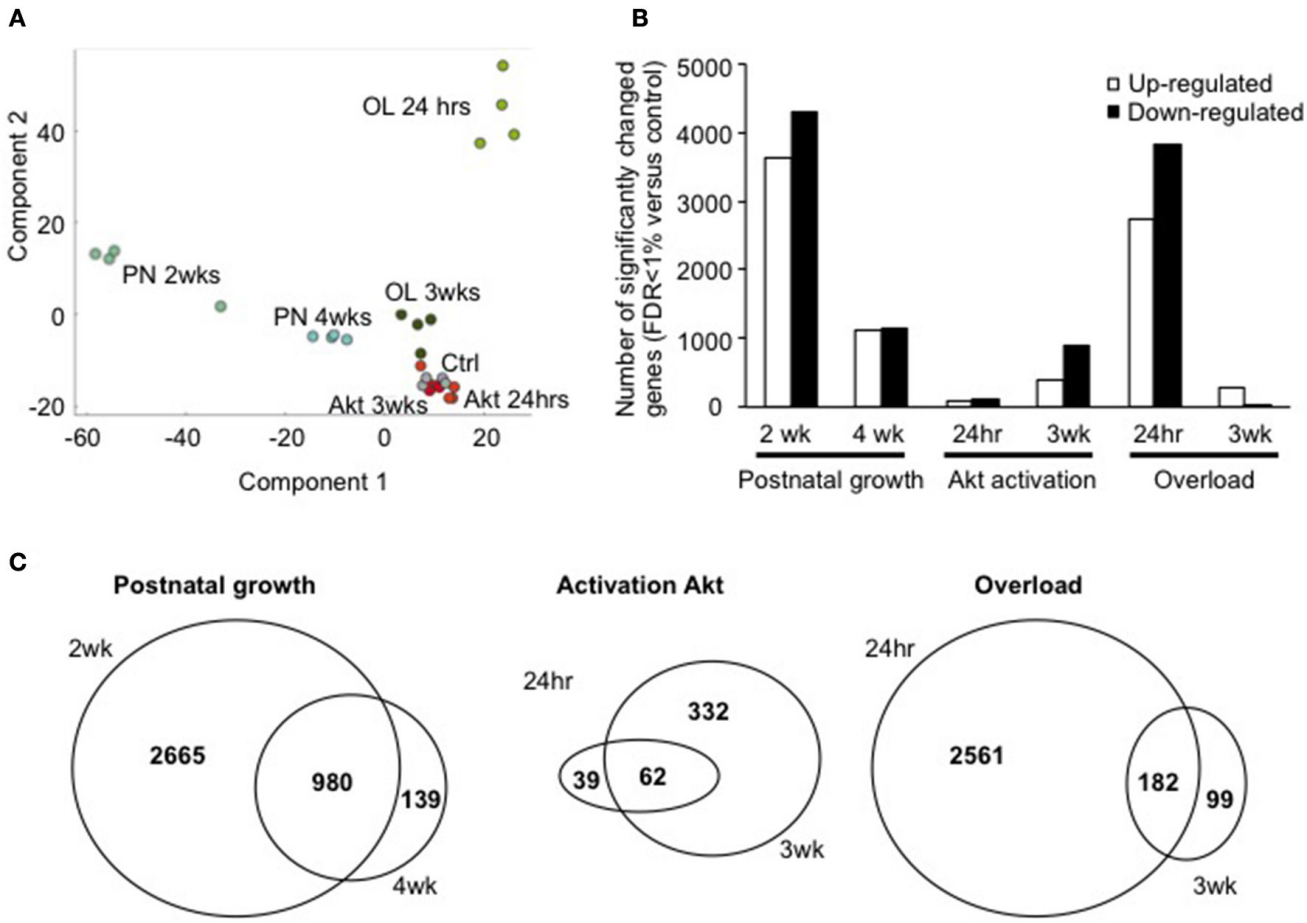
The number of significantly regulated genes varies between growth models. **(A)** Principal component analysis shows the group wise distribution of each individual sample. It can be appreciated how each individual sample clusters with the other samples from the same group, and how each group is distinct from the other **(B)** number of significantly up-and-down-regulated genes in each condition **(C)** overlap between two different time points of the same growth stimulus. A significant amount of overlap exists in postnatal growth (2, 4 weeks), Akt activation (24 h, 3 weeks of activation), and overload hypertrophy due to tenotomy of the TA tendon (24 h, 3 weeks).

In order to characterize each model of hypertrophy according to their respective changes in gene transcription, we performed microarray analyses in each condition. To identify the significantly regulated genes we performed a two-sided *T*-test and considered only those genes at an FDR of <1% that was estimated by a permutation based approach (Tusher et al., 2001). In order to understand the variability of the changes in gene transcription in each different group, we performed a PCA. As can be seen in Figure 2A, the individual samples of each group cluster closely together, with postnatal growth (PN) 2 weeks and 4 weeks showing the highest difference to control muscles within the first component, while in component 2 we found the maximum separation to control for OL 24 h.

Accordingly, the highest number of significantly regulated genes was observed in PN 2 weeks and OL 24 h (Figure 2B). Surprisingly, activation of Akt, which leads to a highly significant increase in protein synthesis rates and muscle growth, was not similarly accompanied by a similar strong alteration in gene transcription, particularly at 24 h after Akt activation. In order to get an idea of the distribution of the fold change for each gene with regards to its statistical significance, we performed volcano plots for each condition. As can be seen in Supplementary Figure 2, the number of significantly regulated genes changed considerably depending on the growth conditions and the different time points.

Next, we compared the common transcriptional changes at the two different time points for each condition. While the number of differentially regulated genes changed depending on the time point, there was some overlap in genes regulated at both time points for each growth condition (Figure 2C). The same observation was made for the significantly down regulated genes (Supplementary Figure 3). Taken together from this we can deduce that a specific gene set is regulated by the type of growth stimulus, while another depends more on the timing of the growth stimulus (immediate response or more established growth).

**Figure 3:**
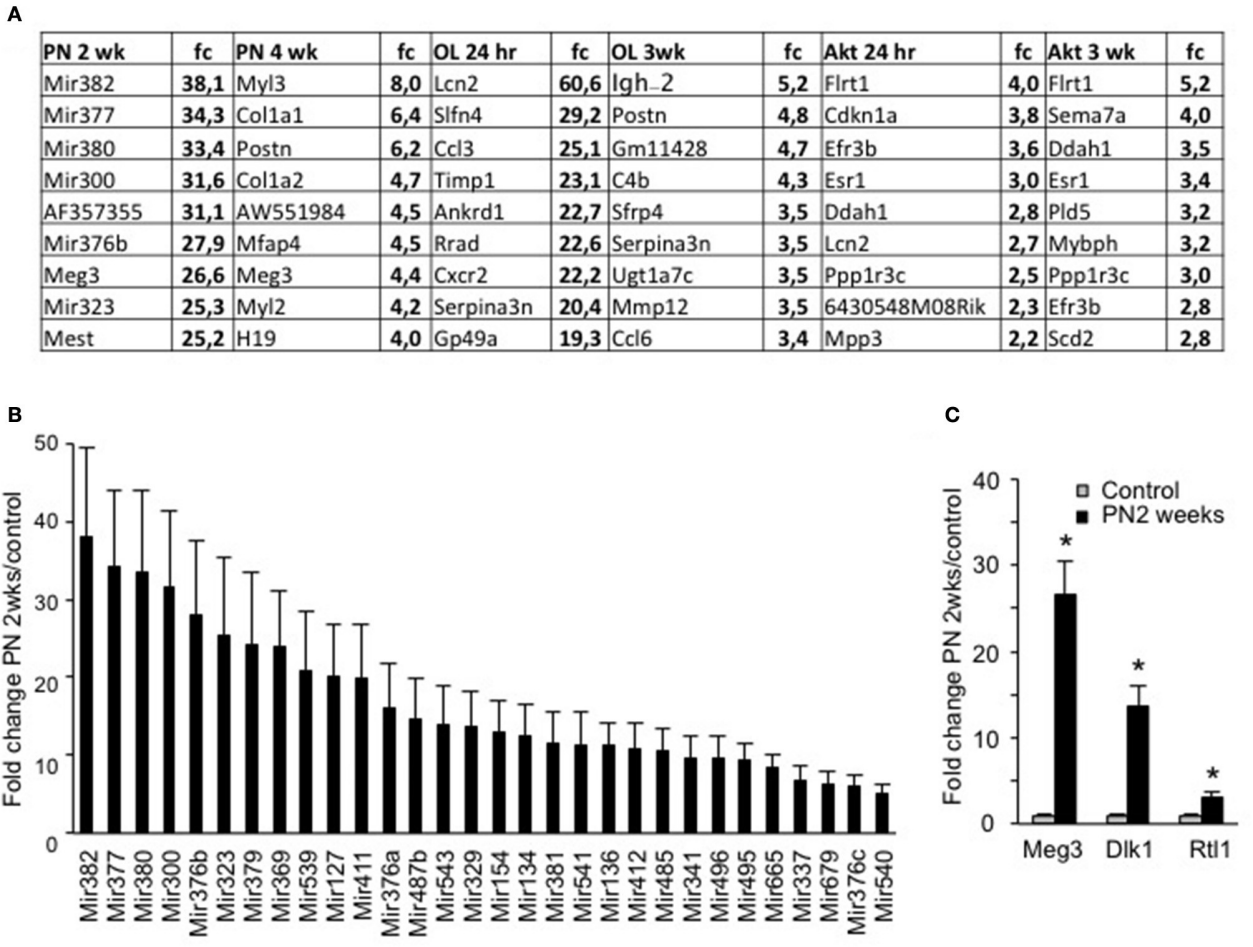
Evaluation of top-ranked genes shows a significant increase in a miRNA megacluster in muscle from postnatal 2 weeks. **(A)** Top ranked significantly increased genes from each condition as compared to control samples. **(B)** Fold change (fc) of 30 miRNAs which showed at least a significant 5-fold change in expression levels in postnatal growth 2 weeks, but in no other condition. **(C)** Fold change of three genes expressed in the same region of the DNA as the miRNA mega cluster, namely the Dlk1-Dio3 locus (*n* = 4 for each group, ^*^P < 0.05).

When we examined the specific genes that showed the greatest differences in expression in each condition (Figure 3A), we found numerous miRNAs with very high fold increases exclusively in the PN 2wks group (Figure 3B). Interestingly, this large group of around 30 miRNAs, which showed a greater than 5-fold increase compared to control mice, all originate from the same mega-cluster of miRNAs localized in the Dlk1-Dio3 locus (Hagan et al., 2009). It has been suggested that transcription of some of these miRNAs is under the control of Mef2A and is required for efficient regeneration (Snyder et al., 2013). With regards to muscle growth, this locus has great interest, since a point mutation in the region between Dlk1 and Meg3 is known to increase their expression levels and cause the hypertrophic muscles of Callipyge sheep (Davis et al., 2004). Importantly, three genes from this locus, Meg3, Dlk1, and Rtl1, all showed a significant increases in PN 2wks EDL muscles compared to control three month old muscles (Figure 3C).

### No common transcriptional program for all models of muscle growth

**Figure 4:**
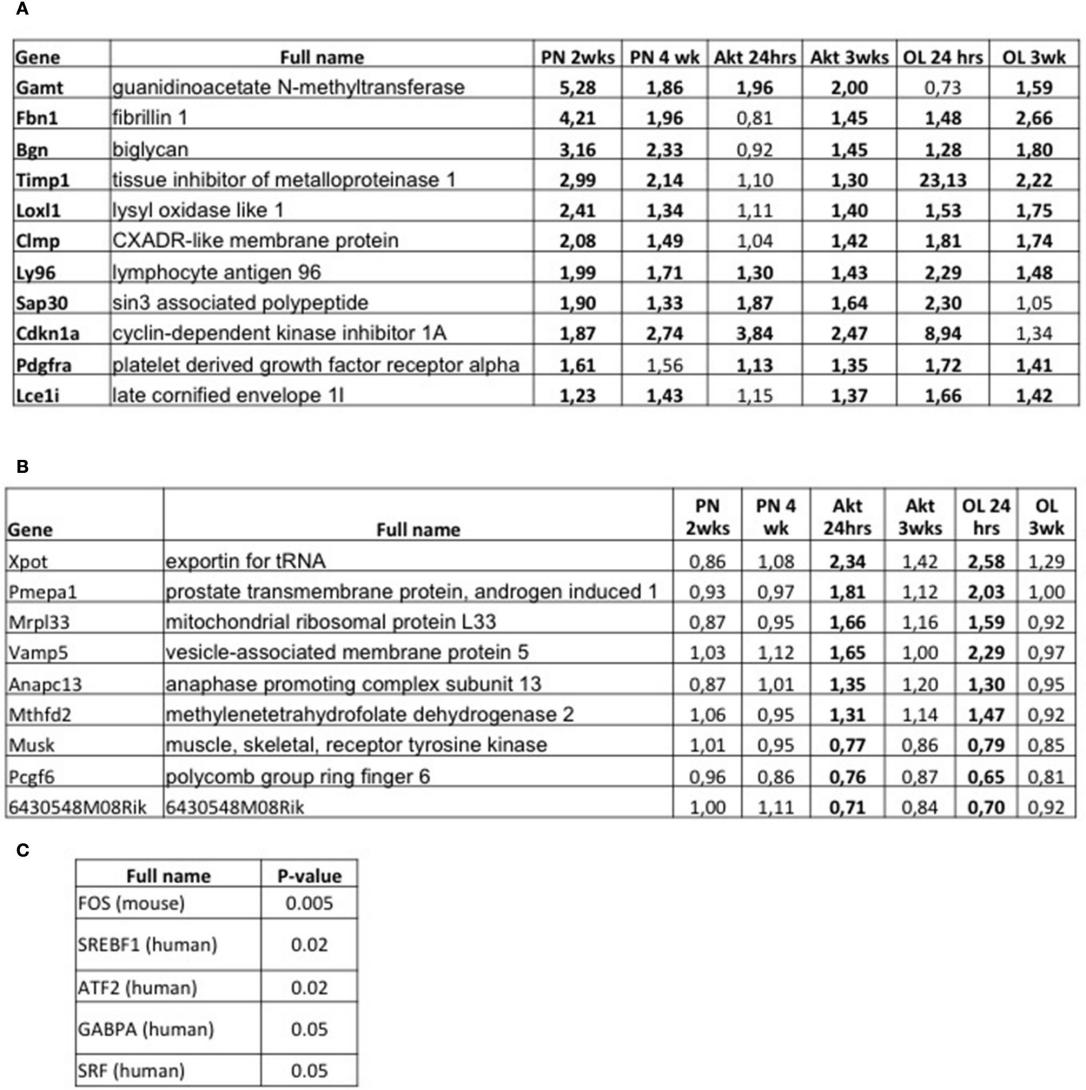
Comparative transcriptome analyses of all models of muscle growth and early signals inducing muscle hypertrophy. **(A)** Table indicating all genes which showed a significant upregulation in at least five of the six growth conditions. Significant changes are shown in bold. **(B)** List of genes which are significantly regulated in Akt 24 h and OL 24 h (in bold), and not in the other growth conditions. These genes are early responsive genes to an acute hypertrophic stimulus. **(C)** TRANSFAC analyses of the genes reported in **(B)** shows a list of five transcription factors whose activity is significantly altered in the early hypertrophic response.

Next, we wondered whether, similar to what was observed for muscle atrophy, there is a common transcriptional program activated in various models of muscle growth. When we considered all genes that show a significant up-regulation in at least five of the six growth models, we identified a list of only 11 genes (Figure 4A). A major reason for this limited amount of genes in common is the fact that there were very few genes differentially regulated in Akt 24 h. Interestingly, we found a higher number of genes which were significantly down-regulated in five out of six models (39 genes, see Supplementary Figure 4). An interesting candidate amongst the upregulated genes was Platelet-Derived Growth Factor Alpha (Pdgfra), recently shown to be required for overload-induced muscle hypertrophy after synergist ablation (Sugg et al., 2017). While the other identified genes are not immediately linked to muscle growth, performing enrichment analyses using the TRANSFAC database, we find that the list of these genes corresponds to an altered activity of three transcription factors, namely TEAD2, CPEB1, and NR1H2 (Supplementary Figure 5A). While the last two are still not well-described in muscle, TEAD transcription factors are well-known mediators of Yap/Taz signaling, which have been linked to muscle hypertrophy (Watt et al., 2015). Performing the same TRANSFAC analyses also for the down regulated genes (Supplementary Figure 4), we find a down regulation of the activity of NR2F1, known to interact with Smad transcription factors and influence TGF-beta signaling (Qin et al., 2013) (Supplementary Figure 5B).

However, since we didn't observe any major transcriptional fingerprint in common to all models, we wondered if there is a gene set which is rapidly activated after an acute hypertrophic stimulus. For this, we analyzed genes which showed a significant regulation only after 24 h of Akt activation or functional overload, but not in all other conditions. This generated a list of only eight genes (Figure 4B), but with some very interesting candidates. One gene (Xpot) is linked to tRNA transport into the cytoplasm, while another is linked to ribosome biogenesis (Mrpl33). Also very interesting was the significant increase in methylenetetrahydrofolate dehydrogenase 2 (Mthfd2), an enzyme involved in the synthesis of purine nucleotides and under the control of mTORC1 (Ben-Sahra et al., 2016). Next, we performed an enrichment analysis to determine transcription factors which are activated in these early moments of muscle growth. As can be seen in Figure 4C, we find a significant increase in the activity of the transcription factors FOS, ATF2, and SRF. Surprisingly, while having a well-documented role in cardiac hypertrophy REF, the role of these transcription factors in inducing skeletal muscle hypertrophy is still an open question.

### Muscle growth is characterized by increased mTOR signaling

**Figure 5:**
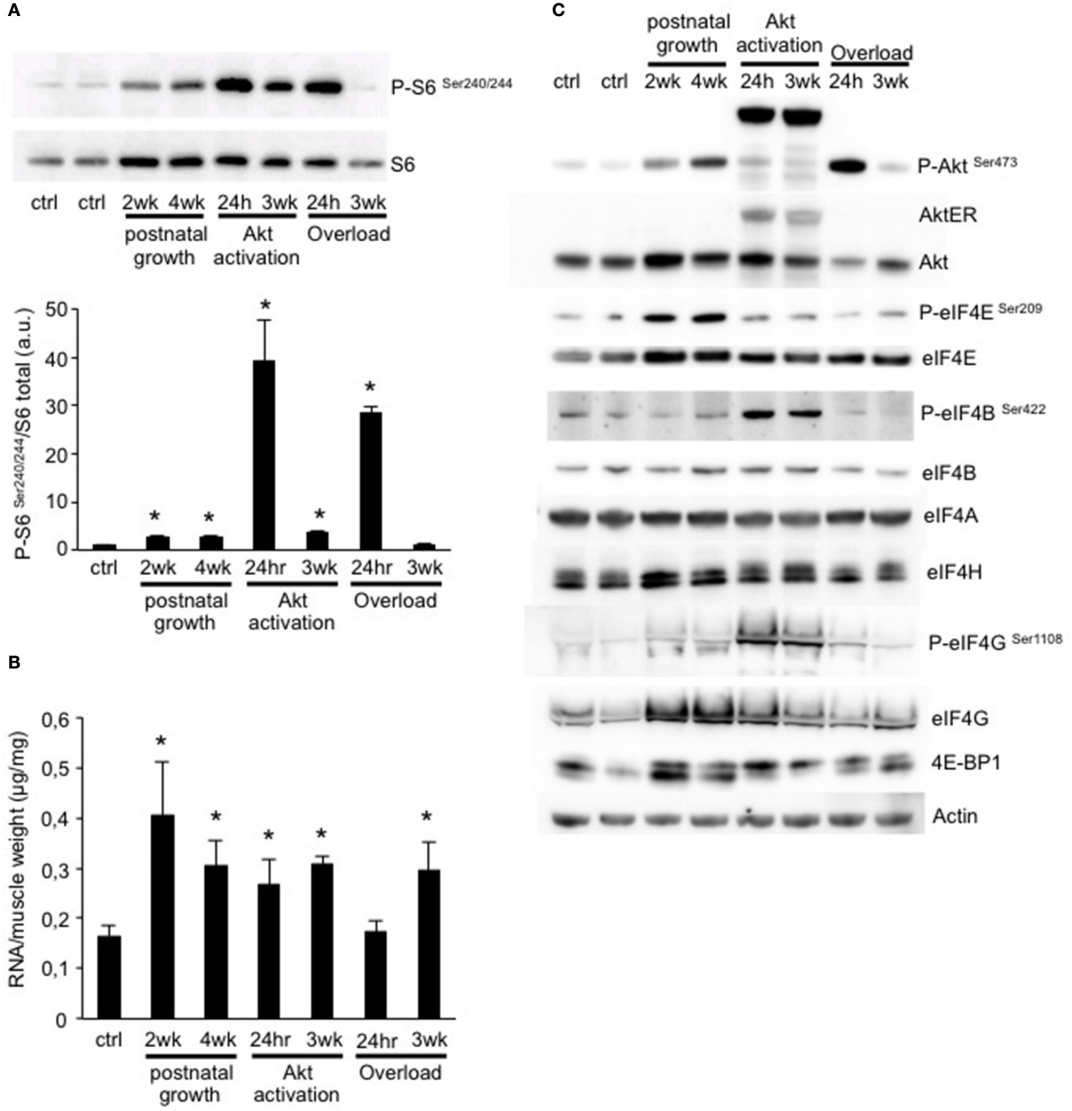
Muscle growth is characterized by an increase in ribosome biogenesis and mTOR signaling. **(A)** Representative blot and quantification of ribosomal protein S6 phosphorylation shows a significant increase in all conditions, except for 3 weeks OL. **(B)** Quantification of the total amount of RNA per muscle weight. All conditions show a significant increase compared to control muscles, except for 24 h OL. **(C)** Representative western blots of the activation of Akt-mTOR signaling and translation initiation factors in all conditions (*P < 0.05, *n* = 4 per group).

While we identified some genes which were regulated in a similar manner in different growth conditions, we didn't observe a major transcriptional program in common to all. However, as all conditions of muscle growth examined show sensitivity to the mTOR-inhibitor rapamycin, we wondered if there might be a translational fingerprint in common between all models. In order to address this we performed a western blot for the phosphorylation status of ribosomal protein S6, a validated marker of increased protein translation. As can be seen in Figure 5A, all conditions showed a significant increase in S6 phosphorylation, except for OL 3weeks. In a recent work we showed that activation of S6K1, the kinase upstream of S6, is required to induce ribosome biogenesis and improve muscle function (Marabita et al., 2016). Interestingly, when we quantified the total amount of RNA normalized for muscle weight, which is a good indicator of ribosome content, we found a significant increase in RNA content in all conditions except for 24 h OL (Figure 5B). The lack of increased RNA/mg muscle in this group is not surprising as muscle weight increases drastically due to the edema formation (Supplementary Figure 1). In order to get a more complete idea of the regulation of Akt-mTOR signaling and the translational apparatus we performed a more extensive analyses by western blotting. As can be seen in Figure 5C, most models show an activation Akt, which nicely mimics the activation pattern of S6 (Figure 5A). Also the inhibitor of cap-dependent translation initiation, 4E-BP1, shows an upward shift, indicative of increased phosphorylation and therefore a reduced inhibition of eIF4E. Furthermore, we find an increase in eIF4E and eIF4G in most models, increasing both the translational capacity as well as reducing the impact of 4E-BP1 inhibition on eIF4E (Thoreen et al., 2012; Siddiqui and Sonenberg, 2015).

## Discussion

Muscle mass is a result of the balance between protein degradation and protein synthesis. More than a decade ago it was shown that protein degradation is characterized by a common transcriptional program in different models of atrophy (Lecker et al., 2004), leading to the identification of a group of genes termed atrogenes. Here, we performed comparative transcriptional profiling in six different conditions of increased protein synthesis, with the aim to identify a common transcriptional program driving muscle growth. While we identified various genes in common between the different conditions, we did not identify a significant number of genes which were regulated in a similar manner during all conditions, i.e., postnatal growth, Akt activation, and overload hypertrophy. On the other hand, when we examined mTOR signaling and translation initiation, we noted that all conditions of muscle growth under investigation were characterized by increased mTOR signaling and ribosome biogenesis, suggesting that different models of muscle growth share a translational, rather than a transcriptional regulation.

In this study, we compared three different models of muscle growth, all of which are characterized by an increase in protein synthesis and muscle force. The fact that all lead to a functional muscle growth makes this study more relevant. Numerous previously reported studies, in which muscle growth was mediated by transcription factors, did not report (Raffaello et al., 2010; Moretti et al., 2016) or did not find (Amthor et al., 2007) an increase in muscle force. Unexpectedly, we found that activation of Akt, which leads to a very rapid increase in muscle mass and function, is not accompanied by a major transcriptional remodeling in the EDL muscle. Previously, we performed microarray analyses on the gastrocnemius muscle (Blaauw et al., 2008; Sartori et al., 2009) and found a significantly higher number of differentially regulated genes 24 and 48 h after Akt activation, which is in accordance with what others observed (Wu et al., 2017). This suggests a different transcriptional regulation in the EDL and gastrocnemius muscles, underlining the importance of comparing transcriptional profiles within the same muscle.

When analyzing the top-ranked genes in each condition, we observed an impressive 30–40-fold increase in multiple miRNAs during postnatal growth 2 weeks. Interestingly, the upregulation of this cluster of miRNAs only occurs in PN 2 weeks and is absent at PN 4 weeks, when satellite cell proliferation has been drastically reduced (White et al., 2010). Interestingly, all these miRNAs originate from the same mega-cluster localized on chromosome 14, in a region called the Dlk1-Dio3 locus. A point mutation in this very complex locus has been shown to be responsible for the hypertrophic muscle phenotype observed in Callypige sheep (Davis et al., 2004). It is thought that this point mutation leads to an increase in the Dlk1 levels, which is influenced by the miRNAs, and sufficient to increase muscle mass. Interestingly, it was found that Dlk1 colocalizes in postnatal muscle with Pax7-positive cells (White et al., 2008), suggesting a role for this locus in satellite cell proliferation. Indeed, mice lacking the transcription factor Mef2A, required for the full induction of the miRNAs in this cluster, show impaired muscle regeneration (Snyder et al., 2013).

While we did not identify a major common transcriptional fingerprint between the different growth models, we did identify some interesting new candidate genes which potentially mediate muscle growth. In the list of genes up-regulated in most conditions, we identified *Pdgfra*, which codes for the Platelett-Derived Growth Factor Receptor Alpha. This receptor is expressed predominantly in muscle-resident stem cells, called Fibro/Adipogenic Progenitors (FAPs), and not in muscle fibers themselves (Uezumi et al., 2011). Interestingly, it was shown recently treating mice with a pharmacological inhibitor of PDGF completely prevents overload-induced muscle hypertrophy (Sugg et al., 2017). Considering its role in angiogenesis, it is tempting to assume that the formation of new vessels through activation of Pdgfra is required for functional muscle growth. In accordance with this is the fact that activation of Akt leads to a significant proliferation of interstitial cells and increased capillarization (Blaauw et al., 2009). In a second, more specific analysis, we filtered for genes which were only significantly regulated after 24 h of overload or Akt activation. This analysis identified 8 genes, which are possibly important first regulators, immediately after a hypertrophic stimulus is given. Some very interesting candidates were identified in this relatively short list. We found an important increase in *Xpot*, a gene responsible for the transport of nascent tRNA from the nucleus to the cytoplasm where the tRNA can participate in protein synthesis. Interestingly, reduction in Xpot levels and subsequent tRNA accumulation in the nucleus lead to a decrease in mTOR signaling (Huynh et al., 2010). Another early response gene was methylenetetrahydrofolate dehydrogenase 2 (*Mthfd2*), a key enzyme regulating purine synthesis in the cell and is under the control of mTORC1. These transcriptional changes linked to altered mTOR signaling correspond nicely to the strong phosphorylation of S6 and 4E-BP1, and the puromycin incorporation, which is most pronounced in these two models of muscle growth. Lastly, performing an enrichment analysis on the total gene list we identified five transcription factors with altered activity levels. Interestingly, of these five factors three are required for the expression of immediate early genes and AP-1 dependent transcription, i.e., Fos, ATF2, and SRF. While Fos activity is known to be linked with cardiac hypertrophy, its role in skeletal muscle hypertrophy has not been properly addressed. Finding a significant increase also in the activity of Serum Response Factor (SRF), which is known to transcribe Fos and Jun, gives further support to an important immediate early gene response in these groups. It is very important to point out that while overload hypertrophy is accompanied by significant damage in these early stages, this is not the case for Akt activation, suggesting this activation of Fos/ATF2/SRF is not a damage response. If and how immediate early genes contribute to muscle hypertrophy in skeletal muscle is an intriguing and open question, which requires further investigation.

An important role of mTORC1 signaling and increased translation initiation during muscle growth is suggested by the strong inhibitory effect of the mTORC1-inhibitor rapamycin in most models of muscle growth. However, in order to inhibit mTOR signaling, rapamycin forms a complex with FKBP12, which also has important roles in the regulation of the Ryanodine Receptor (RyR) (MacMillan and McCarron, 2009). Furthermore, it was shown that rapamycin only blocks some downstream mediators of mTORC1 (Kang et al., 2013), raising the question of which potential mTOR targets mediate muscle growth? In this study we show that increased phosphorylation of ribosomal protein S6 is a key marker of muscle growth. Only in OL 3weeks did we not find any increase in phosphorylation of S6, but this might be due to the fact that a growth plateau had been reached. We recently showed that the S6K1, a major rapamycin-sensitive mTORC1 target, is required for ribosome biogenesis during muscle growth (Marabita et al., 2016). Here, we see that, indeed, an increase in mTOR signaling nicely corresponds to an increase in total RNA levels when normalized for muscle mass. Due to edema formation in the 24 h OL group, we did not see an increase in this ratio, in agreement with a previous report (Chaillou et al., 2015). In order to assess more extensively mTOR signaling and translation initiation, we performed multiple western blots examining key proteins and protein modifications involved in translation initiation. Besides an increase in the phosphorylation of the canonical mTORC1 target 4E-BP1 (as evidenced by the upward shift of the bands), we also find an important increase in eIF4E protein levels in all groups. This is very suggestive of an increased translation initiation, as the ratio of eIF4E/4E-BP1 is critical in determining the amount of cap-dependent translation-initiation (Alain et al., 2012). Taken together, our results suggest that mTOR signaling and translation initiation are the key processes in common between these six different models of skeletal muscle growth.

From this comparative analyses we can conclude that mTOR signaling, ribosome biogenesis, and translational regulation all increase in different models of skeletal muscle growth. These results, together with the known inhibitory effect of rapamycin on muscle growth, make it very tempting to suggest that most models of muscle growth depend on mTOR. It should be pointed out, however, that while rapamycin can reduce multiple models of muscle growth, in numerous cases it does not completely block muscle growth (Marabita et al., 2016). Furthermore, some transgenic models with rapamycin-insensitive hypertrophy have been described (Raffaello et al., 2010). Accordingly, a more in-depth analysis of the importance of mTOR signaling on muscle growth, and its specific functions required for increasing muscle mass and force is warranted.

## Ethics statement

Animals were handled by specialized personnel under the control of inspectors of the Veterinary Service of the Local Sanitary Service (ASL 16—Padova), the local officers of the Ministry of Health. All procedures are specified in the projects approved by the Italian Ministero Salute, Ufficio VI (authorization number 5/2013 PR).

## Author contributions

MP, KD, and BB designed the experiments; MP, KD, LN, FS, MM, MB, FC, EG, HN, and VR performed the experiments; MP, KD, LN, FS, MM, MB, FC, EG, HN, and VR performed data analysis; BB provided scientific expertise; MP, KD, and BB wrote the manuscript.

### Conflict of interest statement

The authors declare that the research was conducted in the absence of any commercial or financial relationships that could be construed as a potential conflict of interest.

## Supplementary material

Supplementary Figure 1 Related to Figure 1. H&E staining of EDL muscles at 24 h and 11 days after tenotomy of the TA tendon. Significant spaces (edema) are observed between the fibers 24 h after tenotomy and are absent at 3 weeks.

Supplementary Figure 2 Related to Figure 2. Volcano plots of the different growth models show the log fold change against the *P*-value. All significantly regulated genes are shown as blue dots.

Supplementary Figure 3 Related to Figure 3. **(A)** Significantly down regulated genes comparing each growth condition at two different time points. **(B)** Top-ranked down-regulated genes in each condition organized by fold change reduction.

Supplementary Figure 4 Related to Figure 4. List of genes which show a significant downregulation in at least five out of six conditions. Significant changes are shown in bold.

Supplementary Figure 5 Related to Figure 4. TRANSFAC analyses performed on genes which show a significant increase **(A)** or decrease **(B)** in five out of six conditions. This shows which transcription factors show a significant change in their activity for respectively the up-and down regulated genes.
