# Supplementary figures and images for: Comparative Analysis of Muscle Hypertrophy Models Reveals Divergent Gene Transcription Profiles and Points to Translational Regulation of Muscle Growth through Increased mTOR Signaling
Source: Front Physiol. 2017 Dec 4;8:968. doi: 10.3389/fphys.2017.00968 (PMC5723052; doi:10.3389/fphys.2017.00968)

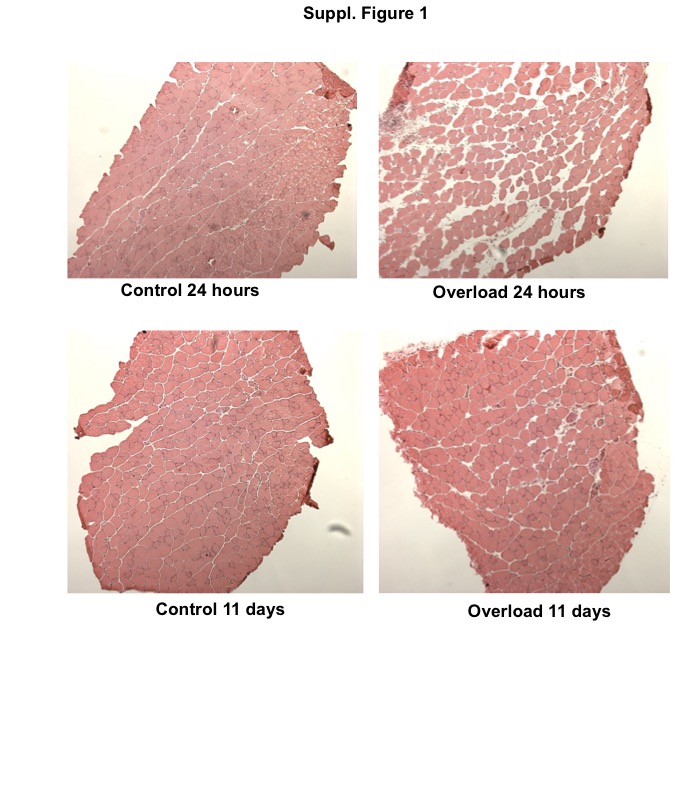

Supplement: Supplementary Figure 1 — Related to Figure 1. H&E staining of EDL muscles at 24 h and 11 days after tenotomy of the TA tendon. Significant spaces (edema) are observed between the fibers 24 h after tenotomy and are absent at 3 weeks. [file Image1.JPEG]

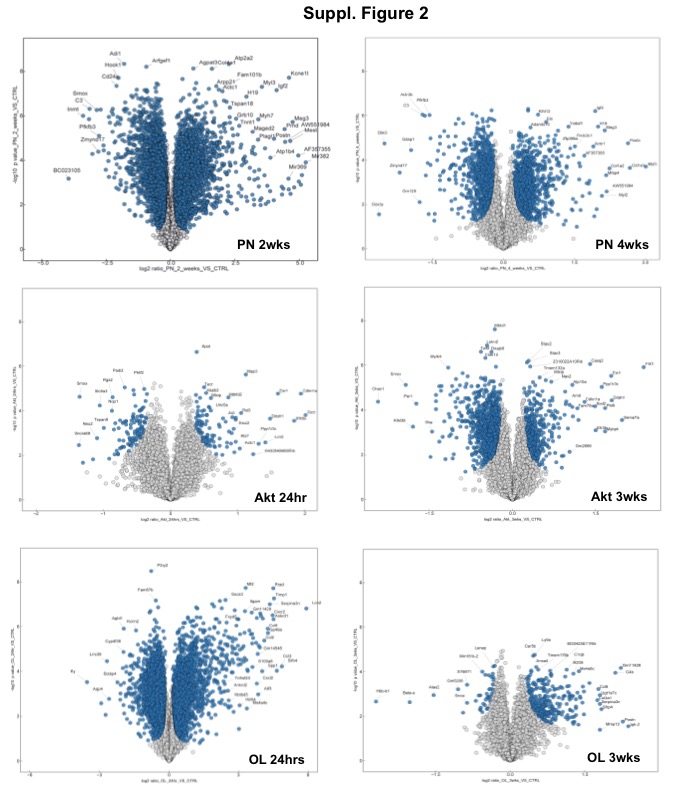

Supplement: Supplementary Figure 2 — Related to Figure 2. Volcano plots of the different growth models show the log fold change against the P-value. All significantly regulated genes are shown as blue dots. [file Image2.JPEG]

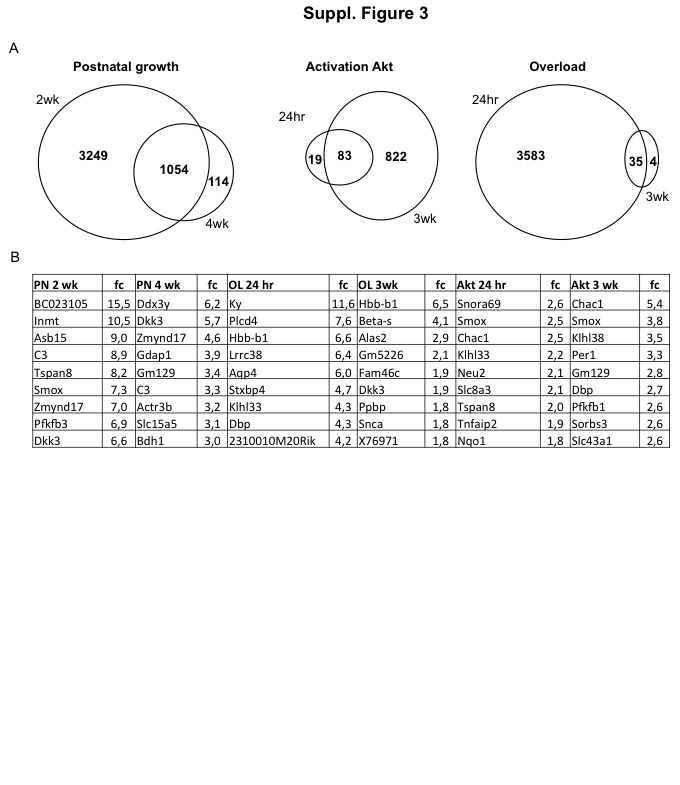

Supplement: Supplementary Figure 3 — Related to Figure 3. (A) Significantly down regulated genes comparing each growth condition at two different time points. (B) Top-ranked down-regulated genes in each condition organized by fold change reduction. [file Image3.JPEG]

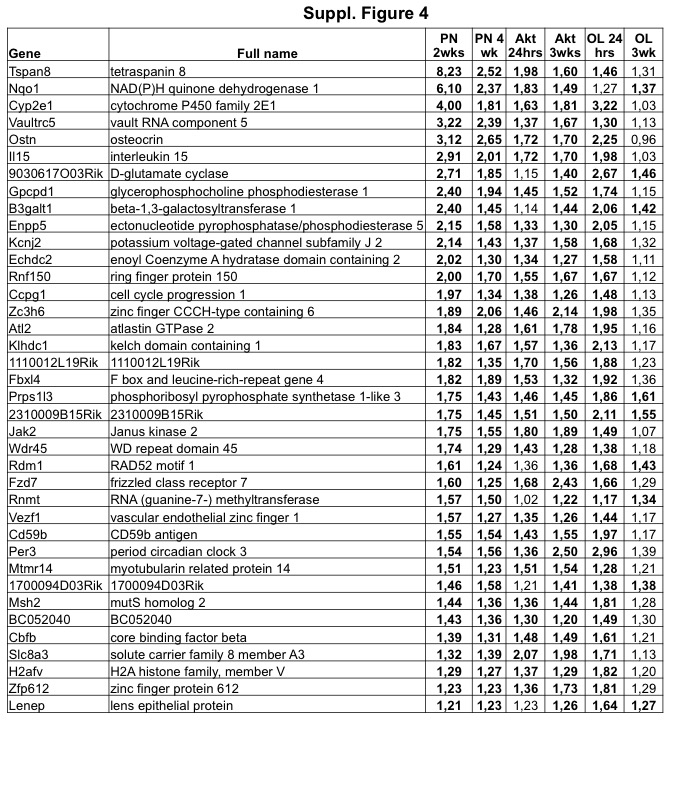

Supplement: Supplementary Figure 4 — Related to Figure 4. List of genes which show a significant downregulation in at least five out of six conditions. Significant changes are shown in bold. [file Image4.JPEG]

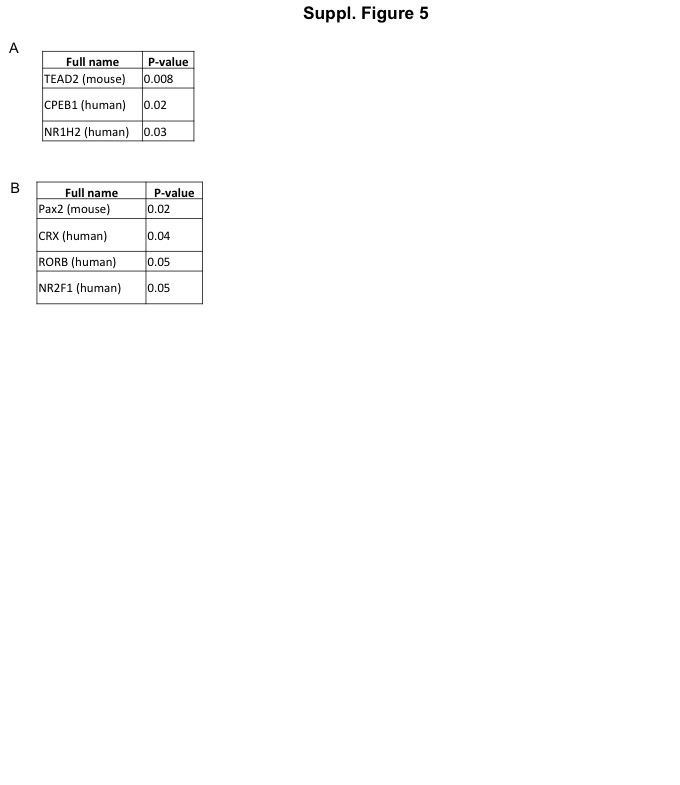

Supplement: Supplementary Figure 5 — Related to Figure 4. TRANSFAC analyses performed on genes which show a significant increase (A) or decrease (B) in five out of six conditions. This shows which transcription factors show a significant change in their activity for respectively the up-and down regulated genes. [file Image5.JPEG]
